# An empirical study of indoor air quality in badminton stadiums in hot summer and cold winter regions of China during spring and fall seasons

**DOI:** 10.1038/s41598-024-53996-z

**Published:** 2024-02-10

**Authors:** Lin Liu, Yong Ma, Ruifeng Huang, Shijie Lin, Mengyao Jia, Gan Liu, Weitao Zheng

**Affiliations:** 1 R&D Testing Sharing Platform for Harmful Substances in Sports Venues of Hubei Province, Wuhan Sports University, Wuhan, 430079 China; 2 Engineering Research Center of Sports Health Intelligent Equipment of Hubei Province, Wuhan Sports University, Wuhan, 430079 China; 3 Key Laboratory of Sports Engineering of General Administration of Sport of China, Wuhan Sports University, Wuhan, 430079 China; 4 Department of Physical Education, Northwest Polytechnical University, Xi’an, 710072 China

**Keywords:** Hot summer and cold winter zone, Badminton hall, Indoor air quality, Field study, Climate sciences, Environmental sciences, Environmental social sciences

## Abstract

The indoor air quality has a direct impact on human health. In order to obtain the current status of indoor air quality in typical sports buildings in hot summer and cold winter climate zones in China, indoor badminton courts in 10 cities in Hubei Province in this climate zone were selected as research objects for field testing of indoor environmental parameters in spring and autumn, and predict air quality parameters for non-testing times. All the tested stadiums are naturally ventilated in non-event conditions, and the average daily indoor CO_2_ concentration was 526.78 ppm in spring and 527.63 ppm in autumn, and the average daily PM_2.5_ concentration was 0.035 mg/m^3^ in spring and 0.024 mg/m^3^ in autumn, all of which met the requirements of GB/T 18883-2022, the average concentration of CO_2_ ≤ 1000 ppm and PM_2.5_ ≤ 0.05 mg/m^3^. The indoor CO_2_ concentration and PM_2.5_ concentration of the tested badminton halls under natural ventilation gradually increased with the accumulation of exercise time, making the indoor air quality of the badminton halls decrease, which would negatively affect the health of the people exercising in this environment.

## Introduction

People spend a large part of their lives and work indoors, and indoor air quality (IAQ) is directly related to human life and health^1^. In recent years, with a large number of building materials, decorations and furniture into the indoor environment, the concentration and types of indoor environmental pollutants in buildings have increased significantly, indoor air pollution has intensified, and IAQ has seriously decreased, resulting in various "Sick Buildingnd Syromes" (SBS), which seriously threaten human health. Recent statistics show that the number of deaths caused by air pollution is about 8.8 million worldwide^2^, therefore, indoor environment quality (IEQ) in buildings has become a high concern today. Currently, common indoor environmental pollutants include carbon dioxide (CO_2_), carbon monoxide (CO), ozone (Ozone, O_3_), particulate matter (PM), formaldehyde (HCHO) and total volatile organic compounds (TVOC)^3^.

In the 1970s, awareness of the seriousness of IAQ pollution began to increase, and therefore research on IAQ began to increase. Some studies have shown that human exposure to indoor pollution may be five times longer than exposure to outdoor^4,5^, which poses a significant risk to human health. The level of indoor CO_2_ concentration reflects the comprehensive level of indoor hazardous gases to a certain extent, and since CO_2_ is easy to measure, CO_2_ concentration is used as an important indicator to test whether IAQ meets the requirements.

China is one of the more serious countries in the world in terms of airborne PM pollution^6,7^, of which the most important pollutant is PM_2.5_ (PM under 2.5 μm in size), which can be inhaled deep into the lungs, and some studies have found that PM_2.5_ that is inhaled deep into the lungs increases bronchitis, the cough and the incidence of lung function defects^8^. In addition, the International Agency for Research on Cancer (IARC) has identified PM as a human carcinogen^9^. Due to the physiological changes that occur during physical activity, people who exercise may be more susceptible to the harmful effects of air pollution^10^, where the body's oxygen consumption and pulmonary ventilation increase during exercise. In addition, the increased demand for oxygen leads to an increase in respiratory rate (sometimes up to 10 times) and air flow rate, thus transferring pollutants deeper into the respiratory tract, i.e., the tracheobronchial or pulmonary region^11^.

In addition, exercisers breathe directly through their mouths, thus bypassing the filtration mechanism of the upper respiratory tract, which retains inhaled particles larger than 2 μm in diameter. Under conditions where exercisers breathe directly through their mouths, PM_2.5_ will bypass the forced filtration mechanism of the upper respiratory tract and enter the body directly. In addition, with exercise, the diffusing capacity of the lungs increases, increasing the intake of gaseous pollutants^12^. Exercise in contaminated air conditions may increase the risk of irritation and allergy, sensitization, acute and chronic respiratory diseases, and pulmonary dysfunction^13^.

Indoor air quality has a significant impact on human health because humans live indoors 90% of the time^14^. Likewise, the air quality of indoor exercise environments affects the health of the exercising population even more, because in indoor exercise environments, humans perform high intensity activities, have high body metabolic rates, and have high rates of gas exchange between respiratory action and the environment. Physical activity has beneficial effects on human health, and for many people, the health benefits and satisfaction associated with physical activity can easily lead them to overlook the indoor air quality of stadiums, which is often worse than atmospheric air quality in most stadiums^15^.

The main gaseous air pollutant in sports facilities is CO_2_, which is a natural product of human respiration^16^. The rate of its production depends mainly on the number of people in the room and the level of metabolism, and the increase in human activity enhances the intensity of respiration, which leads to higher concentrations of CO_2_ in sports rooms than in other places^17^. Exercise promotes physical health, and regular physical activity not only contributes to physical and mental health^18^ but is also beneficial for improving learning and work efficiency^19^ As people place more emphasis on physical activity, indoor sports venues that are not affected by weather are becoming more popular among the general public. For sports buildings, the gathering of large crowds can have a significant impact on IAQ and can lead to human exposure to environments with pollutants. At the same time, when the human body is exercising, the respiratory rate and metabolism are enhanced and more air pollutants may be inhaled, and some studies have pointed out that the dose of particulate matter inhaled by the human body during exercise in the gym is six times higher than in the resting state, and a polluted indoor environment can also affect the performance of athletes^20,21^, therefore, ensuring good indoor air quality in sports buildings is particularly important.

And badminton is a highly popular and popular sport, which is not restricted by age, gender, or skill level, and people of different age groups go to badminton courts for recreational, professional training, or amateur competition purposes^22,23^. According to the 2021 China Sports Venue Statistics Survey^24^, there are 179,300 indoor sports venues in China, including 32,700 badminton courts, accounting for 18.23%, and badminton courts maintain a high usage rate throughout the year. Therefore, the comfort level of indoor thermal environment and the health level of air quality in badminton courts in China is a very worthwhile research, especially after the COVID-19 pandemic, people are more and more interested in the relationship between human health and indoor environmental quality, so the IAQ of badminton courts is a very worthwhile research.

IAQ has an important impact on human health, although a large number of studies have focused on residential^25,26^, office buildings^27,28^ education in different climate zones buildings^29,30^ and IAQ in large commercial buildings^31,32^ but there are fewer relevant studies focusing on sports buildings. In China, stadiums will be built in strict accordance with the design code for sports building JGJ 21-2003 and other national standards for design, and after the stadiums are put into use, with the increase in the number of years of use, the status of IAQ in the pavilion?

Is it able to meet the needs of human health? It is an urgent question that many sports fans need to know at present. Therefore, based on this purpose, this study conducted on-site measurements of indoor air quality of badminton stadiums in different cities of Hubei Province in spring and autumn in the hot-summer and cold-winter climate zones to obtain information on the current status of indoor air quality of stadiums in some cities in this climate zone, and based on the pattern of change of the measurement data, the indoor environmental quality of the venue for other time period prediction, and to provide data support for improving indoor air quality of stadiums and strengthening detection and treatment.

## Methodology

### Test subjects

**Figure 1:**
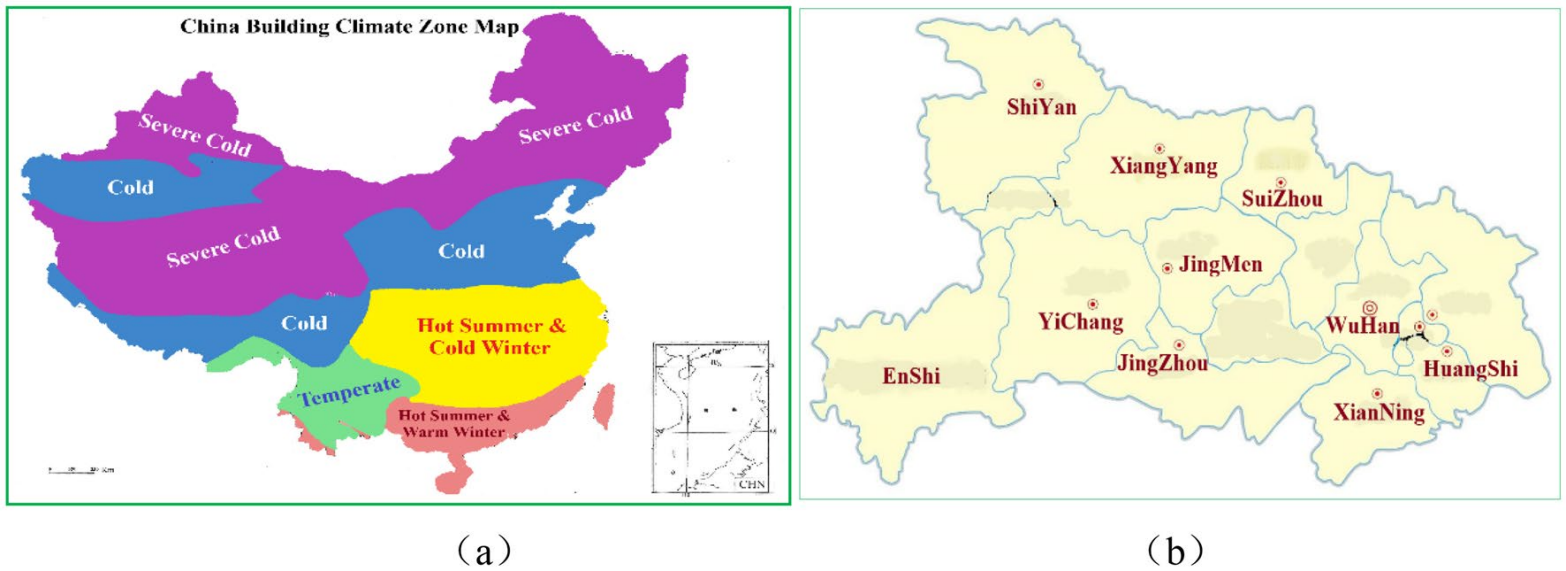
Distribution of test subjects: (**a**) China building climate zone, (**b**) The geographical distribution of test cities.

In the vast country of China, there are five climate zones from north to south, which are severe cold region, cold region, hot summer and cold winter region, hot summer and warm winter region, and mild region. Hubei Province is located in the central part of China, spanning longitude 108° to 116° East and latitude 29° to 33° North, with an east–west length of about 740 km and a north–south width of about 470 km. It is in a subtropical region with four distinct seasons and variable spring and autumn, and is a typical representative of a hot summer and cold winter climate zone, as shown in Fig. 1a. Herein, the cities are selected according to their geographical distribution characteristics and resident populations, and 10 cities are selected for the measurement of IAQ parameters of indoor badminton courts in the end, as presented in Fig. 1b.

The test is planned to be conducted for 14 badminton halls in 10 cities in Hubei Province (including 5 venues in Wuhan), as presented in Table 1, the test venues have demonstrated a long history of badminton; there are venues with special badminton training halls, complexes, and gymnasiums. During the test, all venues are ventilated naturally.

**Table 1:** Badminton courts information.

No	City	Badminton hall	Note
WH1-5	Wuhan	Wuhan Sports University Badminton Hall (WH1), Tongji Medical College Badminton Hall (WH2), Youth Palace Badminton Center (WH3), Wuhan Sports Center Badminton Hall (WH4), Hongshan Gymnasium (WH5)	WH1: space size 2000 m^2^; space height > 9 m; naturally ventilated WH2: built with traditional reinforced concrete; natural ventilation WH3: built with steel-frame structure and glass; simple maintenance structure; natural ventilation WH4: built with traditional reinforced concrete; air conditioning and ventilation system WH5: space size 14,800 m^2^; space height 25 m; air conditioning and ventilation system
XY	Xiangyang	Xiangyang Gymnasium	Space size 20,000 m^2^; space height 30 m; air conditioning and ventilation system
JZ	Jingzhou	Yangtze University Badminton Hall	Naturally ventilated
YC	Yichang	China Three Gorges University Badminton Hall	Built with traditional reinforced concrete; natural ventilation
SY	Shiyan	Shiyan Sports Center Badminton Hall	Space size 3109.13 m^2^; space height > 9 m; naturally ventilated
ES	Enshi	Enshi Nationalities Gymnasium	Space size 7400 m^2^, space height > 9 m, naturally ventilated
JM	Jingmen	Jingmen Sports Culture Center	Space size 16,938 m^2^; space height 32 m; air conditioning and ventilation system
HS	Huangshi	Huangshi Sports Center	Space height 33.71 m; air conditioning and ventilation system
XN	Xianning	Hubei University of Science and Technology Badminton Hall	Built with steel-frame structure and glass; simple maintenance structure; natural ventilation
SZ	Suizhou	New Kangle Badminton Hall	Built with steel-frame structure and glass; simple maintenance structure; natural ventilation

### Test method

**Figure 2:**
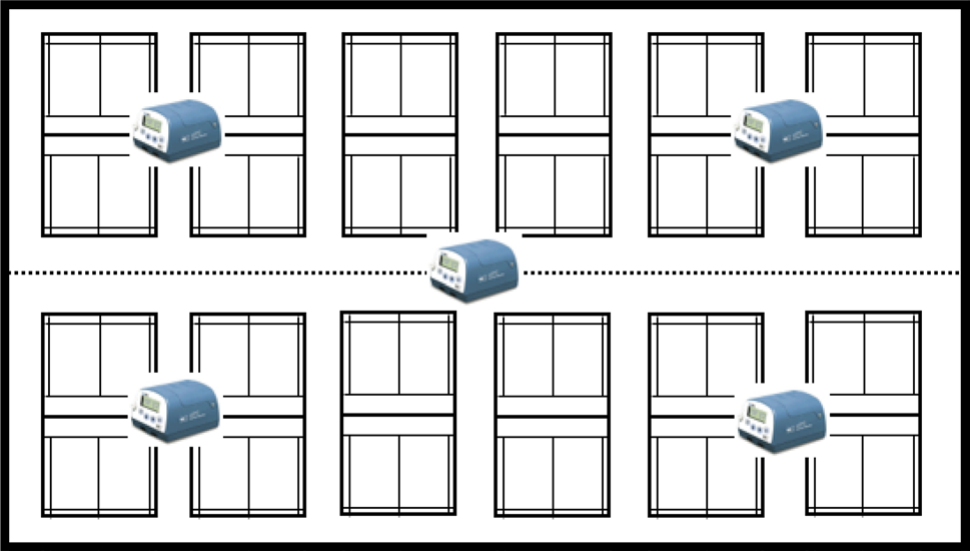
The test location of the badminton courts (5 test points in each hall).

Five equal points on two diagonal lines inside the venue are selected as the test points (see Fig. 2). The test duration for each test point is 5 min and recorded the values after the instrument monitored the stability, and the test is performed every 2 h (Due to the large space of badminton halls, the number of people, exercise area, exercise mode and exercise volume are relatively fixed. Therefore, in this study, the air quality parameters in the arena were selected to be tested at two-hour intervals to investigate the accumulation of quality parameters in the indoor air of badminton halls during a test cycle.). According to ISO7726, the measurement height is about 1.1 m from the ground^33^, and the average value of the five measuring points is taken as the measurement value at each moment. This study was approved by Wuhan Sports University.

### Instruments and evaluation

**Figure 3:**
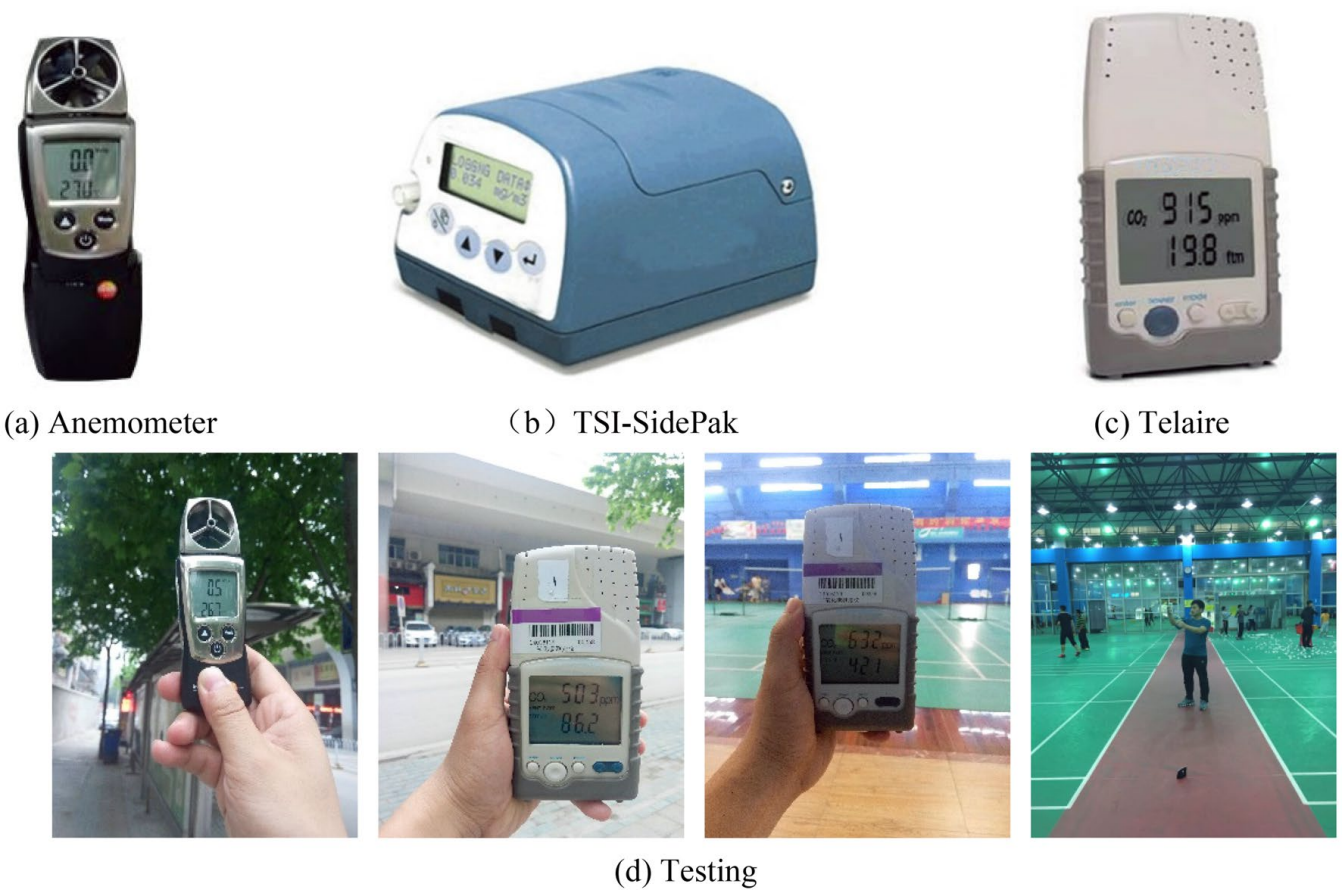
Testing instruments (**a**, **b**, **c**); Testing (**d**).

Indoor environmental parameters measured include air velocity (V), carbon dioxide (CO_2_) concentration, and particulate matter (PM_2.5_) concentration. The specific models and parameters of these apparatuses have been provided in Table 2, the test equipment and test site in Fig. 3.

**Table 2:** Profile of the instrument parameters.

Instrument	Model	Parameters	Range measurement	Accuracy
Anemometer	Testo 410-2	*V*	0.2–20 m/s	0.1 m/s
TSI-SidePak	AM510	PM_2.5_	0.1–10 μm	0.001 mg/m^3^
Telaire	7001	CO_2_	0–10000 ppm	1 ppm

All measured parameters were evaluated according to the existing “Standards for indoor air quality “(GB/T18883-2022) for badminton stadiums in China, as shown in Table 3.

**Table 3:** Evaluation standards.

Standard	*V*	CO_2_	PM_2.5_
GB/T 18883-2022	≤ 0.3 m/s	≤ 1000 ppm	≤ 0.05 mg/m^3^

### Test time

The test duration is set in the range of 10:00 to 20:00, and predictions for data after 20:00. The test team remained in the venue for performing measurements during the closing time at noon, the test is recorded every 2 h. The testing seasons are spring and autumn, with the spring test in early May and the autumn test in late October.

### Ethical review

This study was an on-site test of indoor air quality in badminton halls and did not involve any human experimentation.

### Institutional review board statement

This study was approved by Wuhan Sports University，this research is carried out in accordance with the regulations of the university's research office.

### Informed consent

The tester in Fig. 3-(d) is me (First author), and I agreed to publish the image in an online open-access publication. All participants in this program provided informed consent for participation and for the images to be published online.

## Results and discussion

### Time-varying number of people

**Figure 4:**
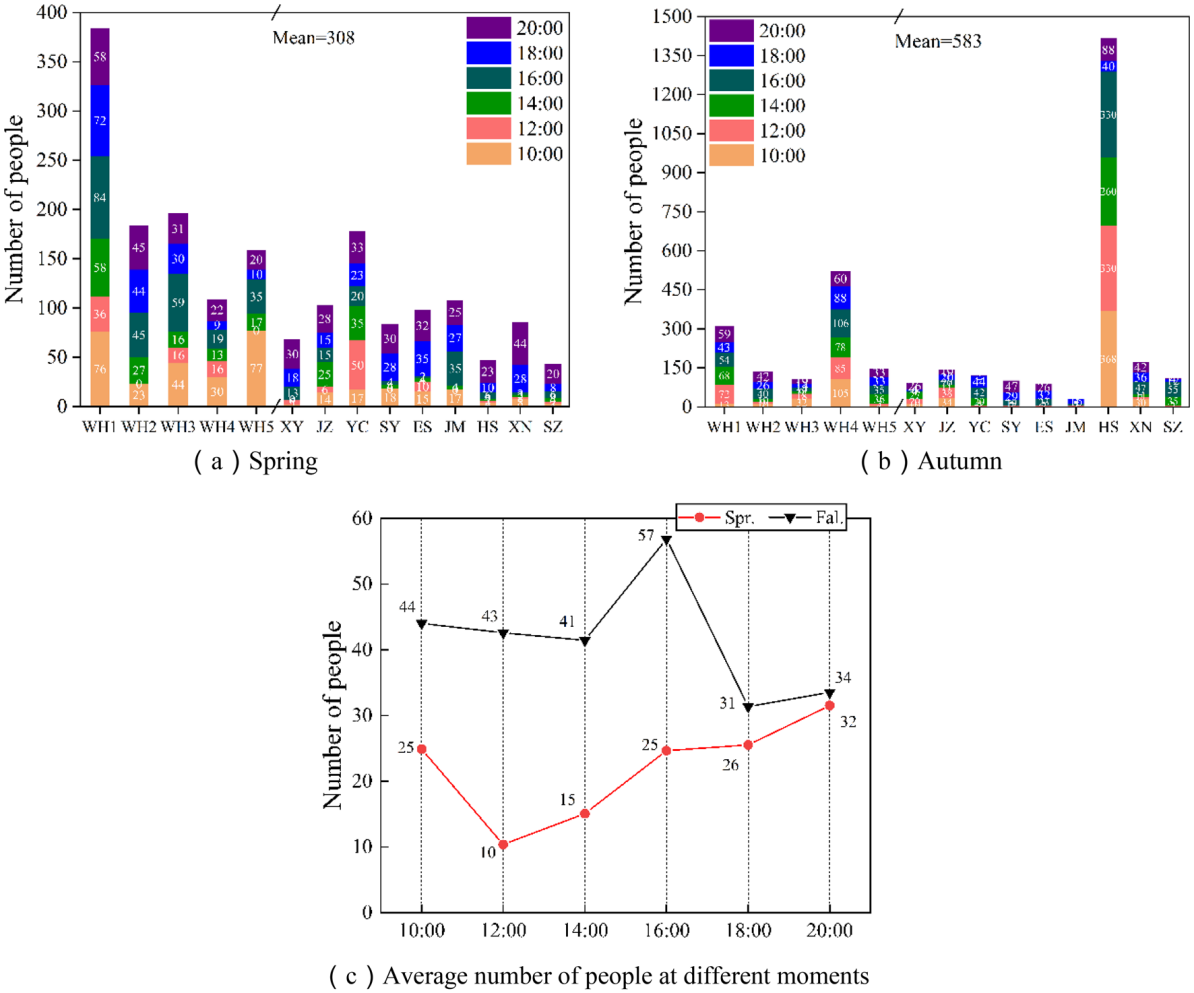
Statistics on the number of people in 14 test venues in different seasons and at different times.

In the test of IAQ parameters, statistics of the number of badminton players in the badminton hall at six different times, as shown in Fig. 4, the sum of the daily average number of people in 14 venues in spring was 308 and 583 in autumn. Since the Huangshi Sports Center holds badminton competitions in autumn, more people play in the venue (the ventilation and cooling system is not turned on during the tournament). From the statistical distribution pattern of the number of people at various instants, the number of people in the venue is generally higher at four moments: 10:00, 16:00, 18:00, and 20:00, Fig. 4C shows that the number of people in the pavilion continued to increase from 12:00 in the spring, reaching 32 people at 20:00, and combining the characteristics of people's working hours and exercise time, it is predicted that the number of people exercising will continue to increase from 20:00 to 22:00, and the growth in the number of people exercising and accumulation of exercise duration will have a considerable impact on the IAQ factors of the badminton hall^34^.

### Wind speed

**Figure 5:**
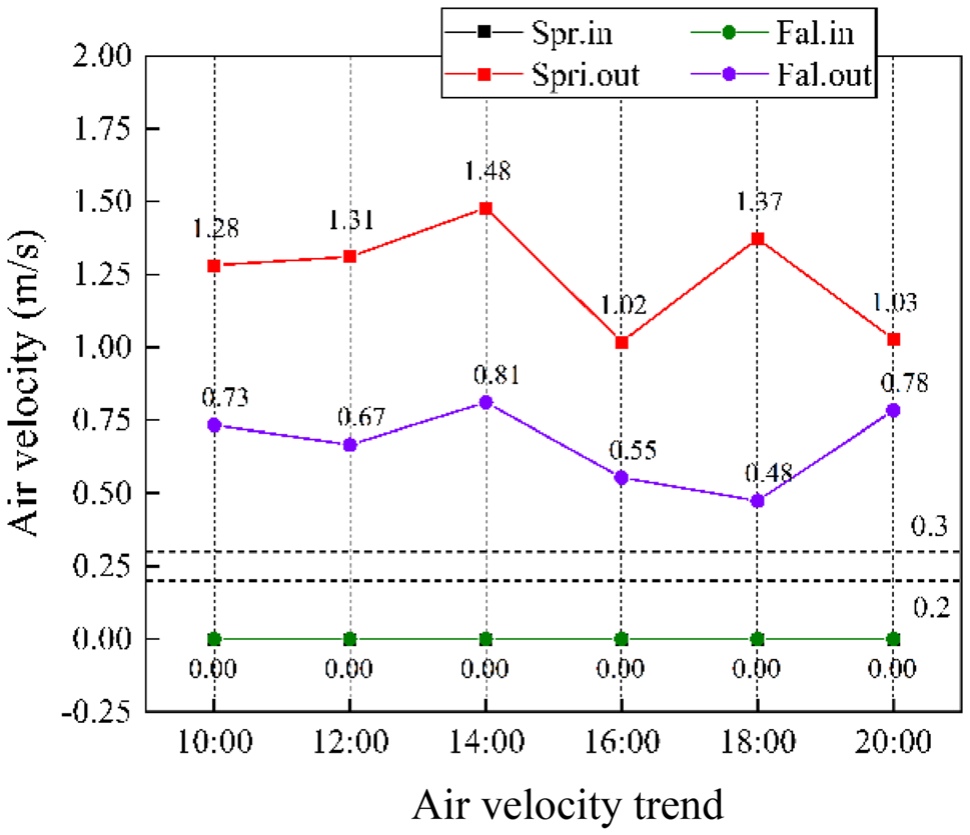
Indoor (outdoor) Air velocity range distribution of badminton courts in different seasons in 14 venues.

As shown in Fig. 5, in the three measured seasons, the outdoor wind speed is higher in spring than in autumn, with an average wind speed at 1.24 m/s, and the average outdoor wind speed in summer and autumn is 0.67 m/s. As shown in Table 4, the indoor wind speed is 0 m/s in spring and autumn. Through the test of wind speed, it was found that all badminton stadiums indoor basically in a windless state, or wind speed less than the range of the test instrument Testo 410-2, in accordance with the requirements of the Olympic badminton stadium wind speed less than 0.2 m/s, all venues tested in terms of wind speed to meet the requirements of badminton tournament sports.

**Table 4:** Statistical summary of indoor environmental parameters and comfort-related indices.

Parameters	Spring			Autumn		
	Mean	Max	Min	Mean	Max	Min
V	0	0	0	0	0	0
CO_2_	526.78	860	386	527.63	1000	407
PM_2.5_	0.035	0.07	0.01	0.024	0.1	0.001

The average values of the measured parameters in different seasons are evaluated for 14 venues, and the mean, maximum, and minimum values of each parameter in all the tested venues in that season are presented.

Analysis from the perspective of IAQ, combined with on-site research found that, because the arena doors and windows are in the closed state, the average daily outdoor wind speed of 1.25 m/s in spring and 0.67 m/s in autumn, spring and autumn outdoor wind speed did not have an impact on the indoor air flow in the badminton hall, so the pavilion air age, IAQ is poor, not conducive to indoor and outdoor air exchange rate and has certain impact on the health of the sports population.

### CO_2_

Medical studies have shown that CO_2_ concentrations above 5000 ppm in indoor environments are dangerous for the organism, especially for individuals with weak physical functions, and that CO_2_ concentrations at 2000 ppm can cause nausea and also seriously affect people's productivity^4,35^. According to the European standard EN 13779, when the CO_2_ concentration in the indoor environment is below 400 ppm it is considered high air quality, when the CO_2_ concentration is between 400 and 600 ppm it is considered medium high air quality, when the CO_2_ concentration is between 600 and 1000 ppm it is considered medium air quality, and low air quality is above 1000 ppm^36^. As shown in Table 3, the GB/T 18883-2022)^37^ requires a CO_2_ concentration ≤ 1000 ppm.

**Figure 6:**
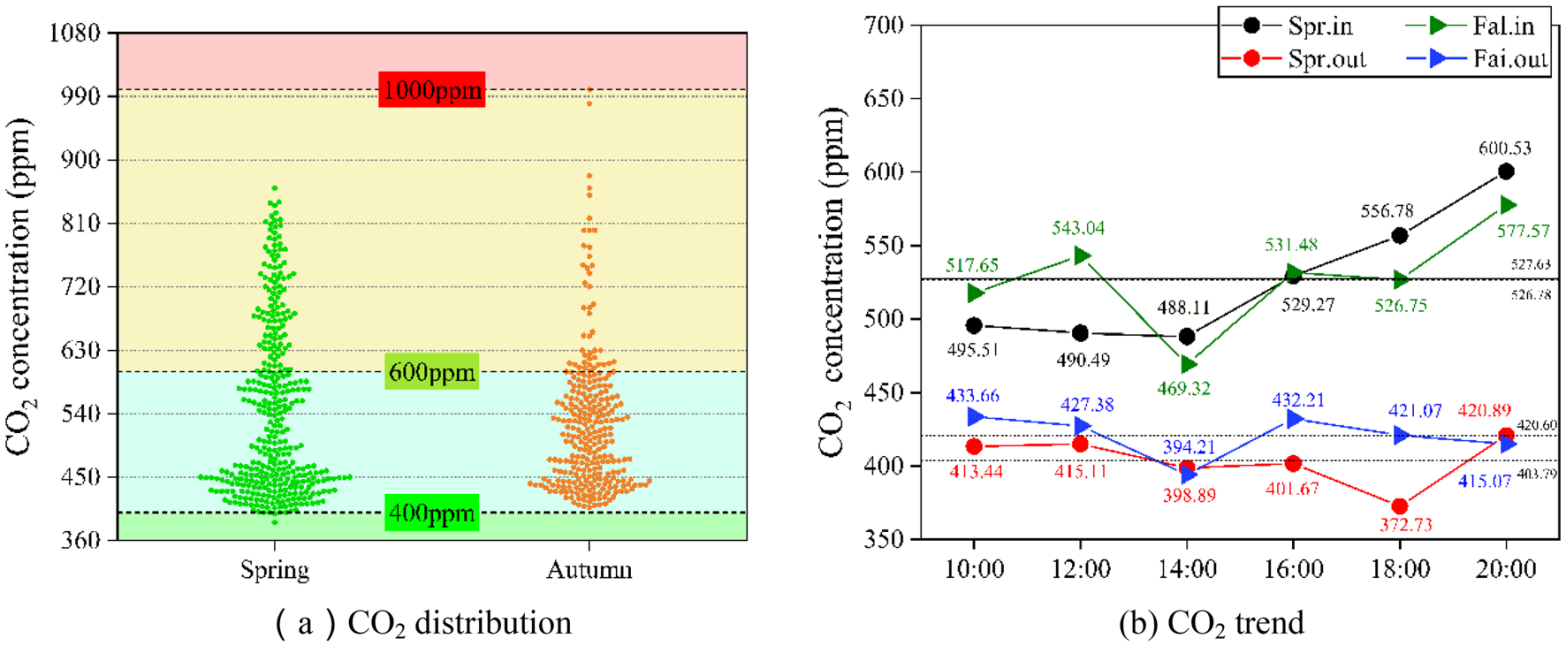
Indoor and outdoor CO_2_ of badminton court in different seasons; (**a**) CO_2_ distribution, (**b**) CO_2_ trend.

As shown in Table 4, in this study, the average indoor CO_2_ concentration in the badminton court was 526.78 ppm in spring and 527.63 ppm in autumn, and the average indoor air CO_2_ concentration was between 400 and 600 ppm in both seasons. According to Nagelkirk et al. the carbon dioxide concentration at a level of 650 ppm ensures acceptable air quality for 90% of the population in indoor stadiums^38^. According to the American Society of Heating, Refrigerating and Air-Conditioning Engineers (ASHRAE), the maximum level of CO_2_ in sports facilities is 1000 ppm^39^, the air quality of the badminton courts tested was of medium to high air quality level. From Fig. 6a, the distribution of CO_2_ concentration at all test points can be found that most of the time, the CO_2_ concentration belongs to the medium to high air quality level of 400–600 ppm. Combined with Fig. 6b from the moment average concentration of measurement statistics, it can be found that the indoor CO_2_ concentration continues to increase in spring and autumn from 14:00, and after 16:00 the indoor CO_2_ concentration is higher than 600 ppm. By 20:00 reached a maximum concentration of 600.53 ppm and 577.57 ppm, respectively, the air quality level is still in the middle and high range, but has reached the critical value of the range, the prediction to 22:00 before the closure of the indoor badminton hall control quality will fall to the middle air quality range, more serious will be in the low air quality range. when CO_2_ concentrations are higher than this value it is considered to be associated with poor air quality, increased incidence of acute health symptoms (such as headaches and mucous membrane irritation), and lower work efficiency^40^.

The outdoor CO_2_ concentration in both seasons has been in the high and medium–high region since 16:00, because of the ventilation, resulting in a gradual decrease in the air quality in the pavilion with the increase in the number of sports people and the accumulation of sports hours. At the same time, based on the characteristics of the measured data in spring and fall and the characteristics of the existing venues, it can be predicted that in the summer with the increase in the number of people playing sports and the enhancement of human metabolism, the oxygen consumption increases, resulting in the indoor CO_2_ concentration will be higher, so that the indoor air quality of badminton stadiums in the summer will be even lower than that of the spring and fall.

As shown in Fig. 4c, the number of people in the badminton court was in a small growth phase during the 8-h period starting from 12:00 to the end of the test, and the increase in personnel led to a decrease in O_2_ concentration and an increase in CO_2_ concentration in the room. And from Fig. 5 ventilation, the average daily outdoor wind speed is 1.25 m/s in spring and 0.67 m/s in autumn, while the indoor measured wind speed is 0 m/s in both seasons.

In the context of the Chinese government's proposed carbon peak and carbon neutral and to meet the premise of basic thermal comfort, reducing energy consumption is an obligation, so the venue's ventilation equipment in the case of non-major events in the unopened state, but due to the strict requirements of the badminton project on wind speed, the doors and windows in the pavilion in the closed state, the indoor and outdoor air exchange rate is low, resulting in a large indoor air age, air quality there is a certain risk of pollution. Also because of the badminton project characteristics, a single field area of 81.74 m^2^ and the pavilion height of not less than 9 m requirements, all badminton indoor space is large enough, even in the absence of mechanical ventilation, the entire indoor air quality within an acceptable range.

### PM_2.5_

**Figure 7:**
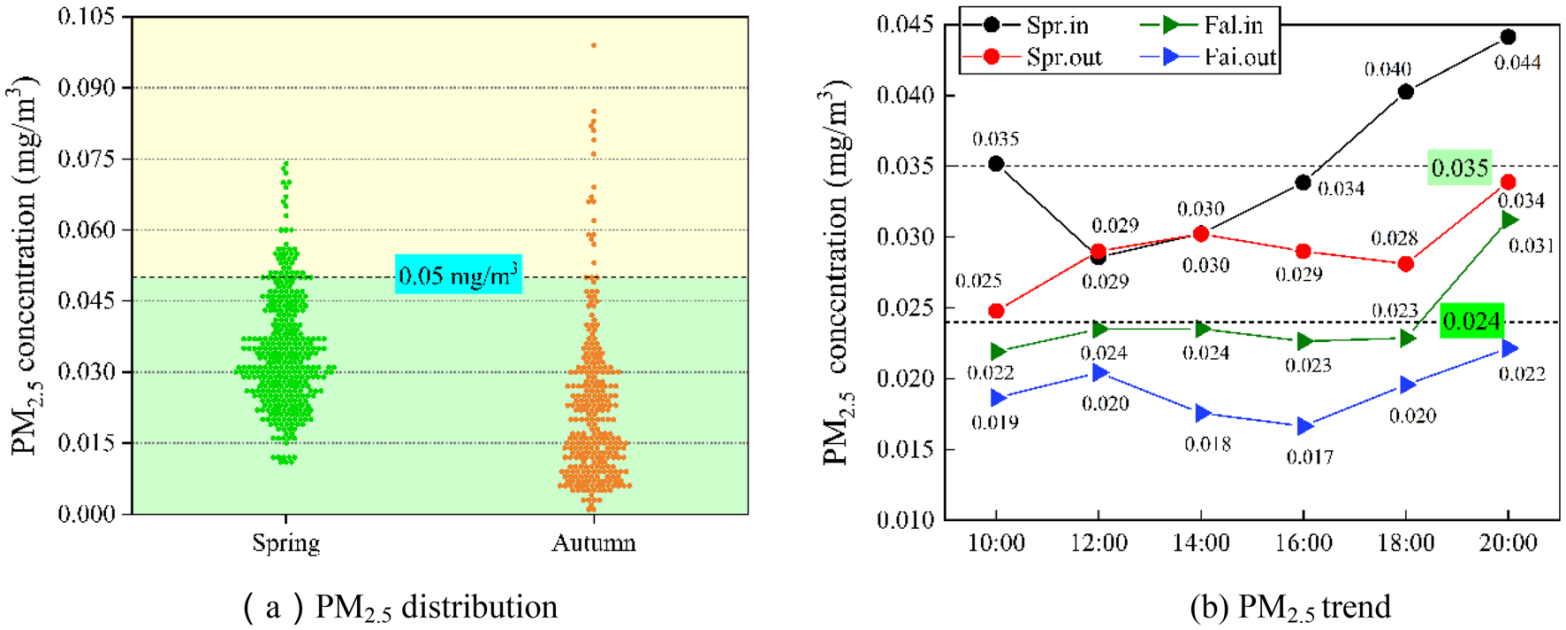
Indoor and outdoor PM_2.5_ of badminton court in different seasons.

As shown in Fig. 7b, the indoor PM_2.5_ concentration in spring varied widely during the test period, with an average concentration of 0.035 mg/m^3^, the lowest concentration value of 0.029 mg/m^3^ at 12:00, and the highest concentration value of 0.044 mg/m^3^ at 20:00. The lowest value of outdoor PM_2.5_ concentration in spring was 0.024 mg/m^3^ at 10:00 and the highest value was 0.034 mg/m^3^ at 20:00; indoor and outdoor PM_2.5_ concentrations in autumn were lower than those in spring, the average indoor concentration in autumn was 0.024 mg/m^3^, the highest PM_2.5_ concentration value was 0.031 mg/m^3^ at 20:00 and the lowest value was The highest PM_2.5_ concentration value was 0.031 mg/m^3^ at 20:00 and the lowest value was 0.022 mg/m^3^ at 10:00, and the highest outdoor PM_2.5_ concentration value was 0.022 mg/m^3^ at 20:00 and the lowest value was 0.017 mg/m^3^ at 16:00 in autumn. As shown in Fig. 7a, the indoor PM_2.5_ concentrations in badminton courts measured in spring and autumn were under the GB/T 18883-2022 national standard (PM_2.5_ ≤ 0.05 mg/m^3^) for most of the time, meeting the health needs of the sports population.

However, because there is no purification device, indoor PM_2.5_ concentration continues to increase with the increase of exercise time, as Fig. 7a In the data obtained from all test points, PM_2.5_ concentration exceeds 0.05 mg/m^3^ in both spring and autumn, and it can also be found from the statistics of Table 4 that PM_2.5_ concentration reaches 0.07 mg/m^3^ at maximum in spring and PM_2.5_ concentration reaches a maximum of 0.1 mg/m^3^ in spring and 0.1 mg/m^3^ in autumn. Unfortunately this study only measured to 20:00, according to Fig. 4c the statistics of the number of people can be predicted that the average concentration of PM_2.5_ from 20:00 to 22:00 will exceed 0.05 mg/m^3^. Through the maximum value of PM_2.5_ concentration in spring and fall statistics in Table 4, it can be found that the maximum value of PM_2.5_ concentration in spring can reach 0.07 mg/m^3^, and the concentration of PM_2.5_ in fall is greater and can reach 0.1 mg/m^3^. Through the maximum value, it can be found that the measured badminton halls in the existing natural ventilation, the concentration of indoor PM_2.5_ will appear to be more than the national prescribed standards. Thus making the badminton court sports environment air quality degradation, the sports crowd to produce some damage.

## Limitations and future challenges

Herein, the following deficiencies exist in this study of the IAQ of 14 badminton courts in 10 cities of Hubei Province in the hot-summer and cold-winter climate region based on the field testing methods:

1. Only the average indoor CO_2_ concentration and average PM_2.5_ concentration in the badminton hall are monitored, and other indicators such as formaldehyde and volatile organic compounds (VOC) are not monitored due to the large number of mats and other mats laid on the floor of the badminton hall, which cannot reflect the IAQ of the badminton hall on the whole.
2. This study only tested the IAQ of badminton courts in the region in spring and autumn, lacking data from summer and winter, which cannot more comprehensively evaluate the current situation of IAQ of badminton courts in this climate zone.

## Conclusion

In this study, a field test study was carried out on the indoor air quality of badminton halls in spring and fall, when the temperature is variable and the cycle is short in hot summer and cold winter climate zones. It was found that the badminton halls in this climate zone were naturally ventilated during the daily opening period, and the test results of CO_2_ concentration and PM_2.5_ concentration showed that the average daily concentration of indoor CO_2_ in spring was 526.78 ppm, the average daily concentration of CO_2_ in autumn was 527.63 ppm, the average daily concentration of PM_2.5_ in spring was 0.035 mg/m^3^, and 0.024 mg/m^3^ of PM_2.5_ daily average concentration in autumn, all of which meet the indoor air quality requirements. Although the daily average value meets the national standard, but through the maximum value of CO_2_ and PM_2.5_ measurement results can be found in the natural ventilation mode, badminton hall indoor air quality will be accumulated with the length of people's sports will appear more than the standard value of the time period, this period of badminton hall indoor air quality is reduced, the health of the sports crowd caused by the negative impact.

This study provides the current status of indoor air quality in typical sports buildings in hot summer and cold winter climate zones in China, and data support for improving indoor air quality of stadiums and strengthening detection and treatment. Only the concentration of PM_2.5_ and CO_2_ in the badminton hall are monitored in spring and autumn. There are not data of PM_2.5_ and CO_2_ for summer and winter and other indicators such as formaldehyde and volatile organic compounds (VOC) are not monitored which cannot more comprehensively evaluate the current situation of IAQ of badminton courts in this climate zone. In future studies, the data of PM_2.5,_ CO_2,_ the formaldehyde and volatile organic compounds (VOC) will be measured within each season.

### Supplementary Information

Supplementary Information.

Supplementary Information.

Supplementary Information.

Supplementary Information.

Supplementary Information.

Supplementary Information.

## Data Availability

All data generated or analysed during this study are included in this published article [and its supplementary information files].
