# Supplementary figures and images for: An empirical study of indoor air quality in badminton stadiums in hot summer and cold winter regions of China during spring and fall seasons
Source: Sci Rep. 2024 Feb 10;14:3427. doi: 10.1038/s41598-024-53996-z (PMC10858893; doi:10.1038/s41598-024-53996-z)

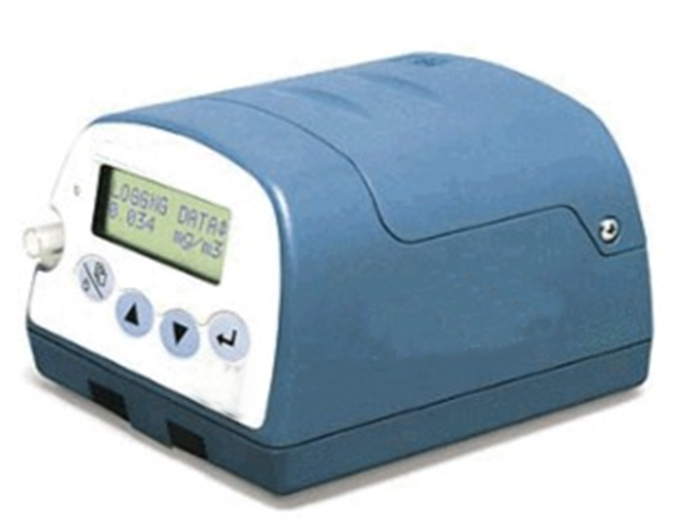

Supplement: Supplementary file 2 — Supplementary Information. [file 41598_2024_53996_MOESM2_ESM.jpg]

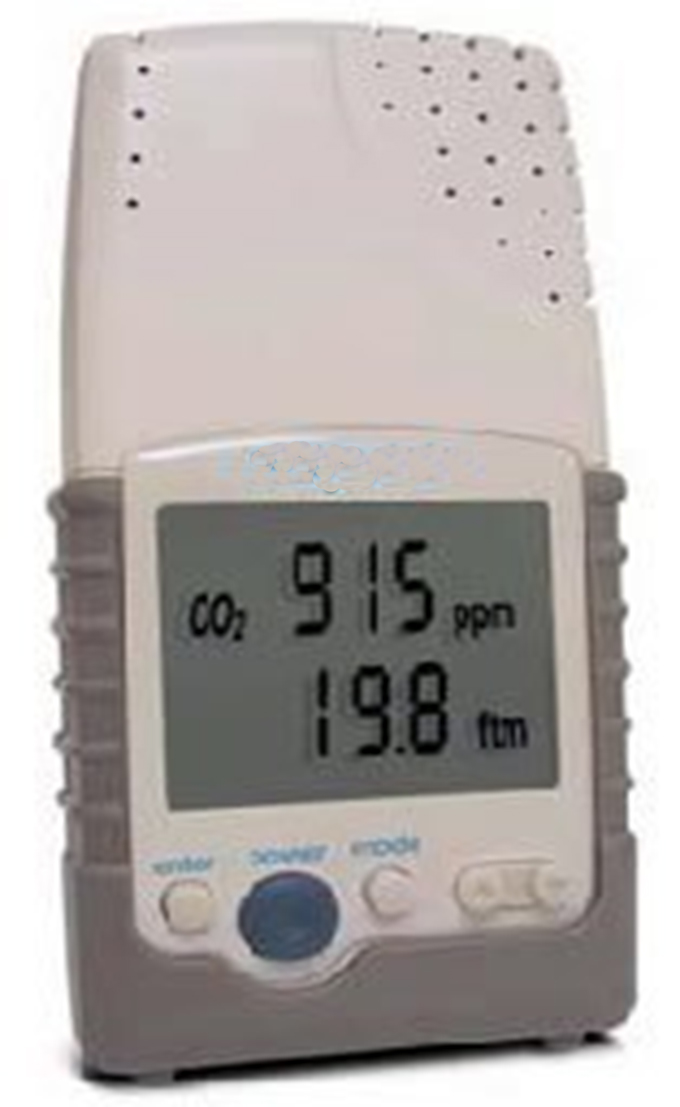

Supplement: Supplementary file 3 — Supplementary Information. [file 41598_2024_53996_MOESM3_ESM.jpg]

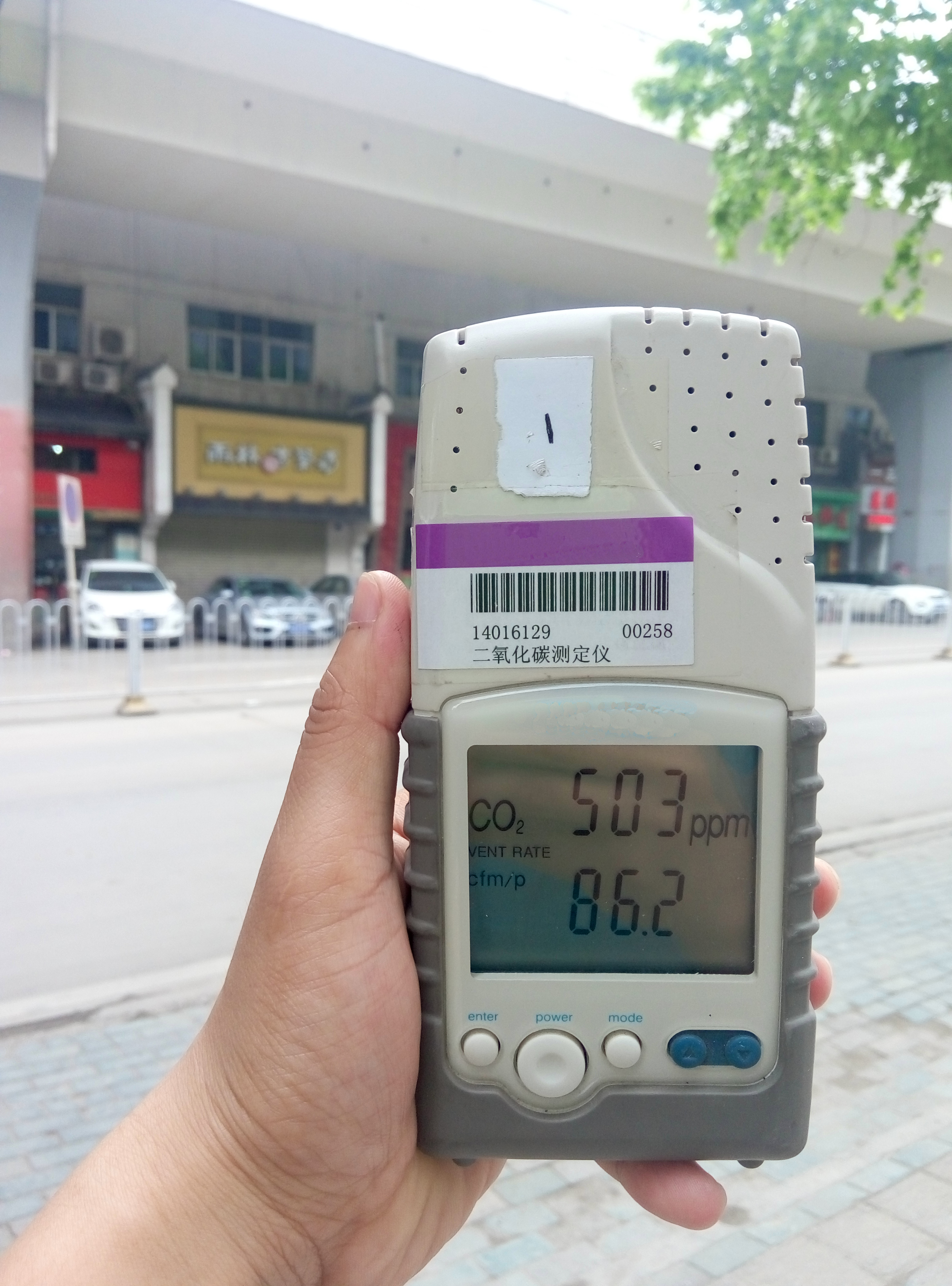

Supplement: Supplementary file 4 — Supplementary Information. [file 41598_2024_53996_MOESM4_ESM.jpg]

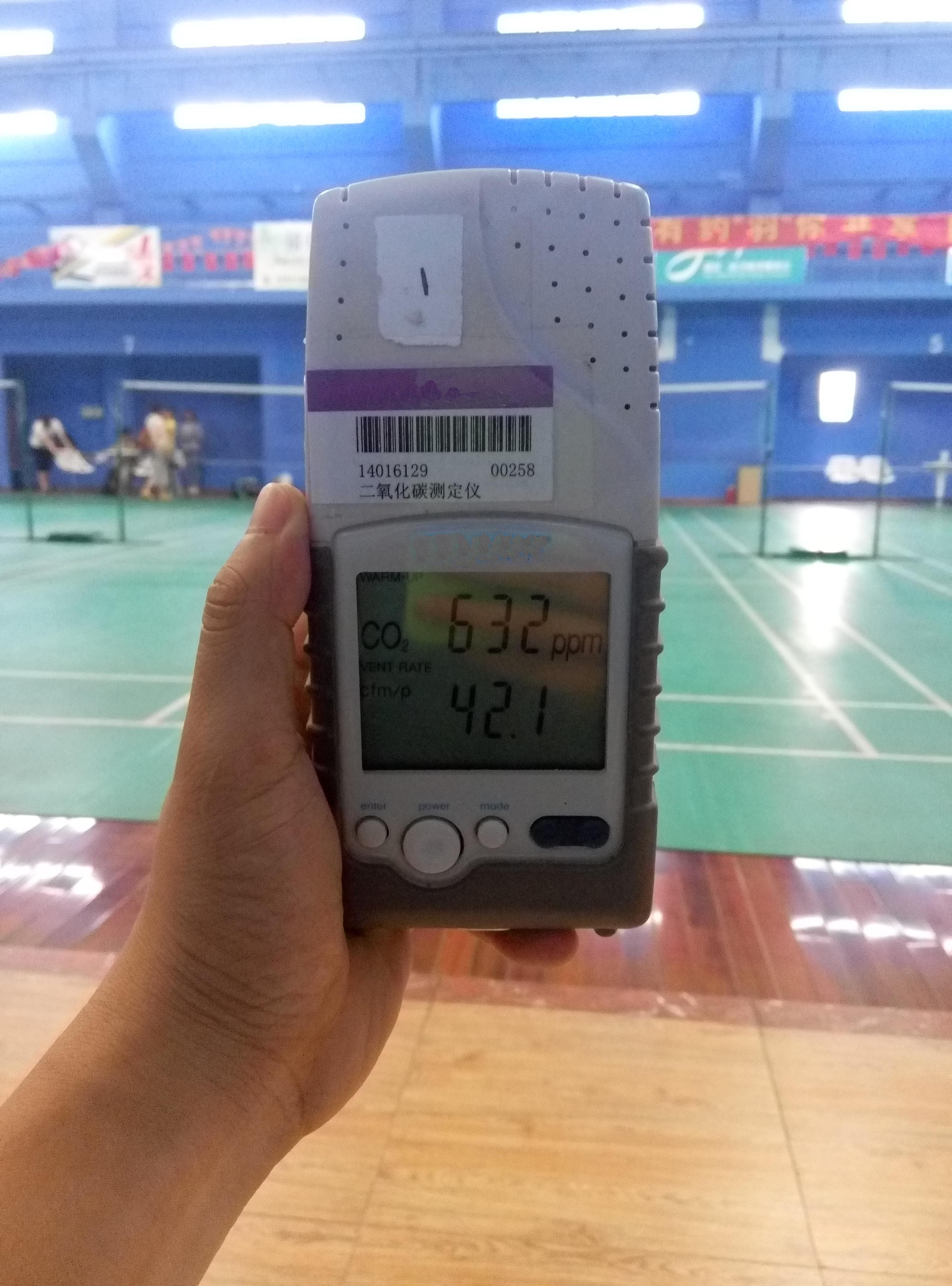

Supplement: Supplementary file 5 — Supplementary Information. [file 41598_2024_53996_MOESM5_ESM.jpg]

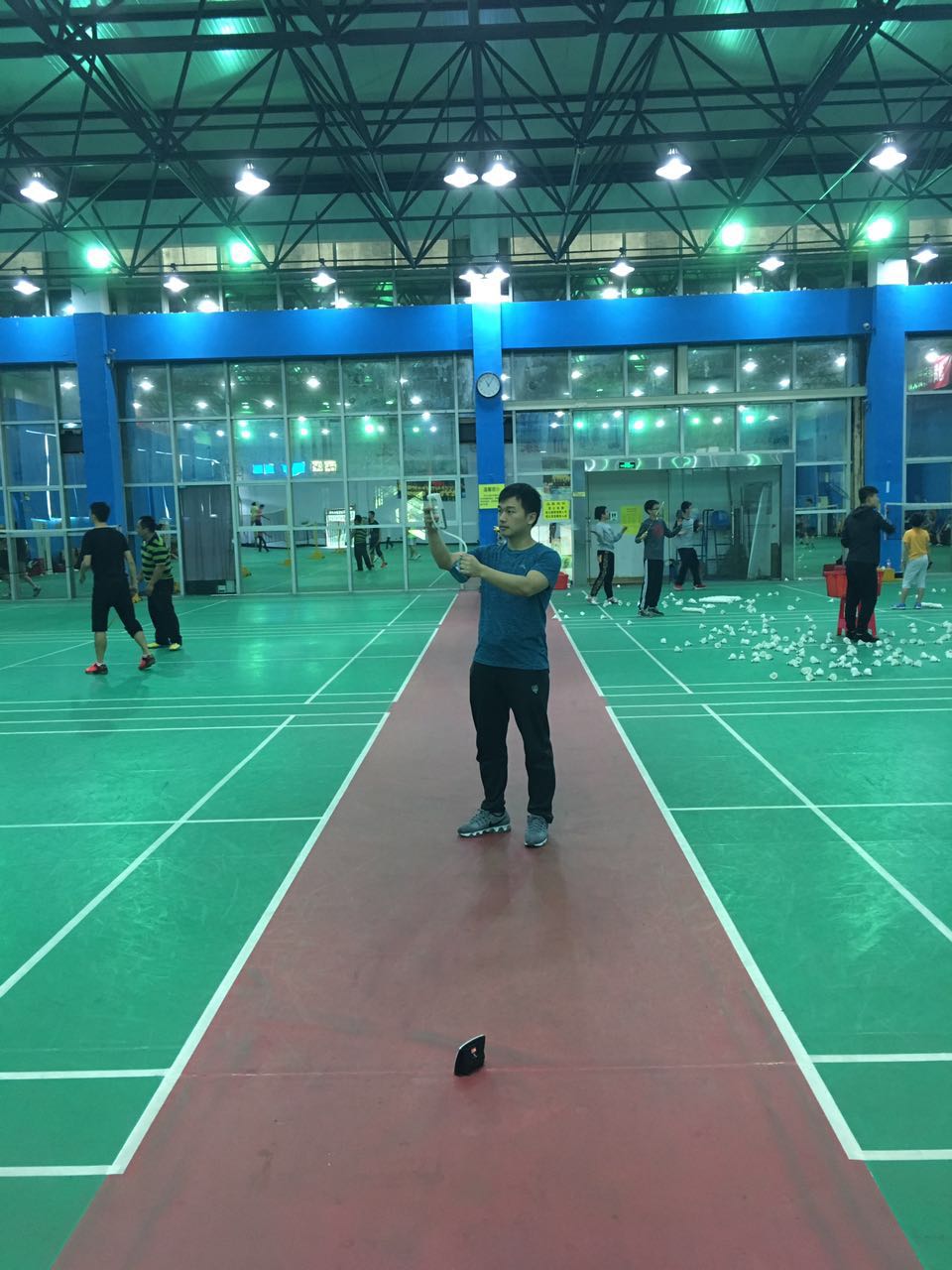

Supplement: Supplementary file 6 — Supplementary Information. [file 41598_2024_53996_MOESM6_ESM.jpg]
